# An unprecedented occult non-communicating rudimentary uterine horn treated with laparoscopic excision and preservation of both fallopian tubes: a case report and review of the literature

**DOI:** 10.1186/s13256-020-02636-x

**Published:** 2021-02-04

**Authors:** G. Gitas, K. Eckhoff, A. Rody, A. K. Ertan, S. Baum, E. Hoffmans, I. Alkatout

**Affiliations:** 1 Department of Obstetrics and Gynecology, University Hospitals Schleswig Holstein, Campus Luebeck, Ratzeburger Allee 160, Haus A, 23538 Luebeck, Germany; 2 Department of Obstetrics and Gynecology, Leverkusen Municipality Hospital, Leverkusen, Germany; 3 Department of Obstetrics and Gynecology, University Hospitals Schleswig Holstein, Campus Kiel, Kiel, Germany

**Keywords:** Müllerian anomalies, Rudimentary uterine horn, Laparoscopic excision

## Abstract

**Background:**

Müllerian duct anomalies are congenital malformations of the female genital tract and may be of various types. For decades they have been classified according to the American Society of Reproductive Medicine, which mentions unicornuate uterine malformations as the second subgroup. They result from the arrested development of one of the Müllerian ducts and appear in approximately 1/1000 women. These anomalies are usually diagnosed in the second decade of life, because they tend to remain asymptomatic until adolescence and their initial symptoms may vary. Patients present with symptoms such as dysmenorrhea, infertility, and chronic or acute abdominal pain.

**Case presentation:**

We report on a 21-year-old Caucasian German patient who suffered from dysmenorrhea for 7 years. After a transvaginal ultrasound and magnetic resonance tomography of the pelvis was performed, the patient underwent a diagnostic hysteroscopy and operative laparoscopy, and was finally diagnosed with a Müllerian duct anomaly presenting with a non-communicating rudimentary uterine horn. The left tube arose directly in orthotopic location from the cornua of uterus, with no connection to the rudimentary uterine horn or structure.

**Conclusion:**

The anatomic features of this case have not been reported previously and were not consistent with any existing classification. More cases are needed in order to confirm our hypothesis. Gynecologists should always consider Müllerian anomalies as an important differential diagnosis in young patients with abdominal pain.

## Introduction

Congenital uterine anomalies are the most common anomalies of the female reproductive system [1]. Organogenesis, fusion, and septal resorption are essential steps for the physiological formation and transformation of the Müllerian ducts. The resorption of midline tissue occurs in the 20th week of gestation, initiating the development of the uterus, the cervix, and the fallopian tubes [2]. The development of these entities may be impaired at any of the abovementioned stages. The production of testosterone and the anti-Müllerian hormone (AMH) in genetic males (46,XY) causes regression of the Müllerian ducts. Therefore, in genetic female embryos (46,XX) the absence of Y chromosomes allows the Müllerian ducts to develop into the abovementioned organs [2].

Seven major categories of anomalies have been defined by the American Fertility Society [3]. Uterine agenesis/hypoplasia or a unicornuate uterus are caused by dysfunction during early organogenesis, resulting in the absence of one or both Müllerian ducts. Failure of canalization causes a unicornuate uterus with a rudimentary horn, which also appears in the early stages of embryogenesis (7-8 weeks of gestation). Furthermore, a bicornuate uterus or didelphys is caused by failure of fusion of the ducts. A septate or arcuate uterus results from subsequent resorption of the central septum (Table 1). An embryological connection has been identified between the Müllerian and Wolffian systems [4]. Therefore, there is a notable coincidence of renal anomalies in patients with congenital malformations of the female genital tract. The presence of renal anomalies in patients with a unicornuate uterus is as high as 40.5% [5]; a one-sided renal agenesis was diagnosed in 28% [6]. Therefore, anomalies of the renal system must be taken into account in patients with Müllerian malformations.

**Table 1:** Correlation between stage of dysfunction of the Müllerian ducts and anomalies

Stage of dysfunction	Müllerian anomalies
Early organogenesis	Uterine agenesis/hypoplasia
	Unicornuate uterus
Failure of canalization	Unicornuate uterus with a rudimentary horn
Failure of the fusion of the ducts	Uterus bicornuate
	Didelphys
Subsequent resorption of the central septum	Septate
	Arcuate uterus

A unicornuate uterus is believed to account for 2% to 13.7% of all uterovaginal anomalies [7]. A non-communicating rudimentary uterine horn was reportedly observed in 7-48% of cases of a unicornuate uterus [8]. Generally, the appearance of a rudimentary horn is rare (0.06%). The rudimentary horn was shown to be associated with a poor reproductive prognosis and a high frequency of cornual pregnancy, endometriosis, and dysmenorrhea.

The treatment usually consists of laparoscopic excision or reconstruction of the affected anatomical structures. Due to the rare incidence of this entity, its diagnosis and surgical treatment remain a challenge. The treatment must be established individually after thorough counseling. Notably, case reports of the experimental treatment of Müllerian anomalies paved the way for the improvement in their diagnosis and surgical treatment [9, 10].

We report on an occult non-communicating uterine horn which has not been mentioned in the known classifications and not described so far in the published literature.

## Case report

A 21-year-old Caucasian German patient had been suffering from chronic secondary dysmenorrhea for 7 years. The patient was hospitalized with ambiguous lower abdominal pain and a suspected parametrial tumor. Her medical history revealed no previous disease or surgery. Since menarche, her menstrual cycles had been regular with moderate flow, but she complained of progressive dysmenorrhea. Conservative treatment with hormonal drugs (combined oral contraceptive pills) was administrated without success for symptom control.

**Fig. 1:**
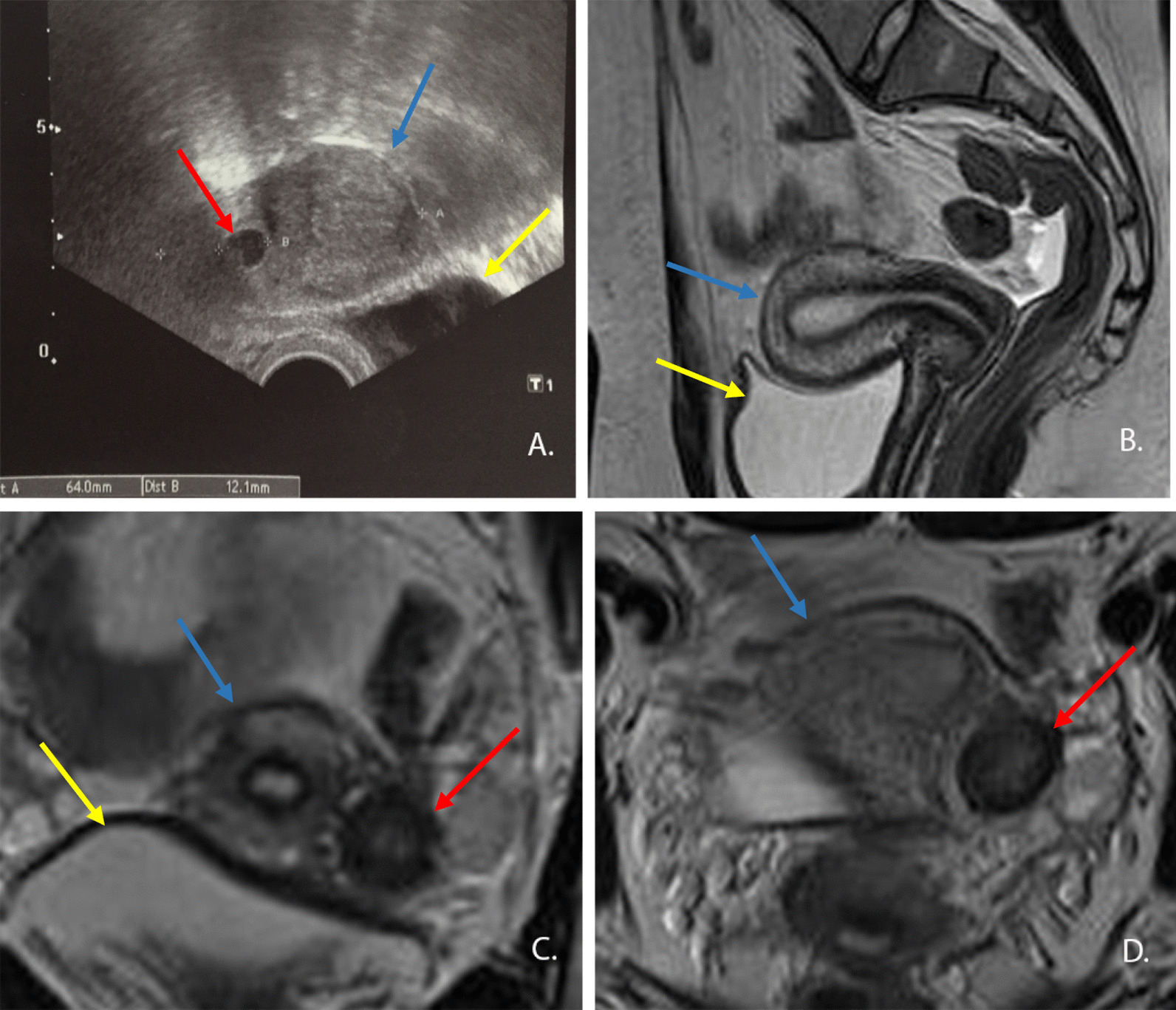
**a** Transvaginal ultrasound in the transverse plane (red arrows mark the rudimentary uterine horn, blue arrow the normal uterus and yellow arrow the bladder). **b** Magnetic resonance tomography (MRT) image in the sagittal plane. **c** MRT image in the transverse plane. **d** MRT image in the frontal plane

The clinical examination, which included inspection and palpation, revealed normal conditions. Transvaginal ultrasound showed an abnormal round structure (Fig. 1a) and a uterus with two cavities. The right cavity was of normal size; the endometrium was also normal. The left cavity appeared to be smaller and the endometrium was distended with blood, similar to hematometra. Both ovaries were normal. The kidney ultrasound was normal. A magnetic resonance tomography (MRT) of the pelvis revealed a uterus with a normal cavity and, to its left, between the uterus and ovary, a structure measuring 2 cm in size with a small central cavity similar to the endometrium, with some residues of blood suggestive of functioning endometrium (Fig. 1b–d). We suspected the presence of a non-communicating uterine horn or an adenomyoma.

**Fig. 2:**
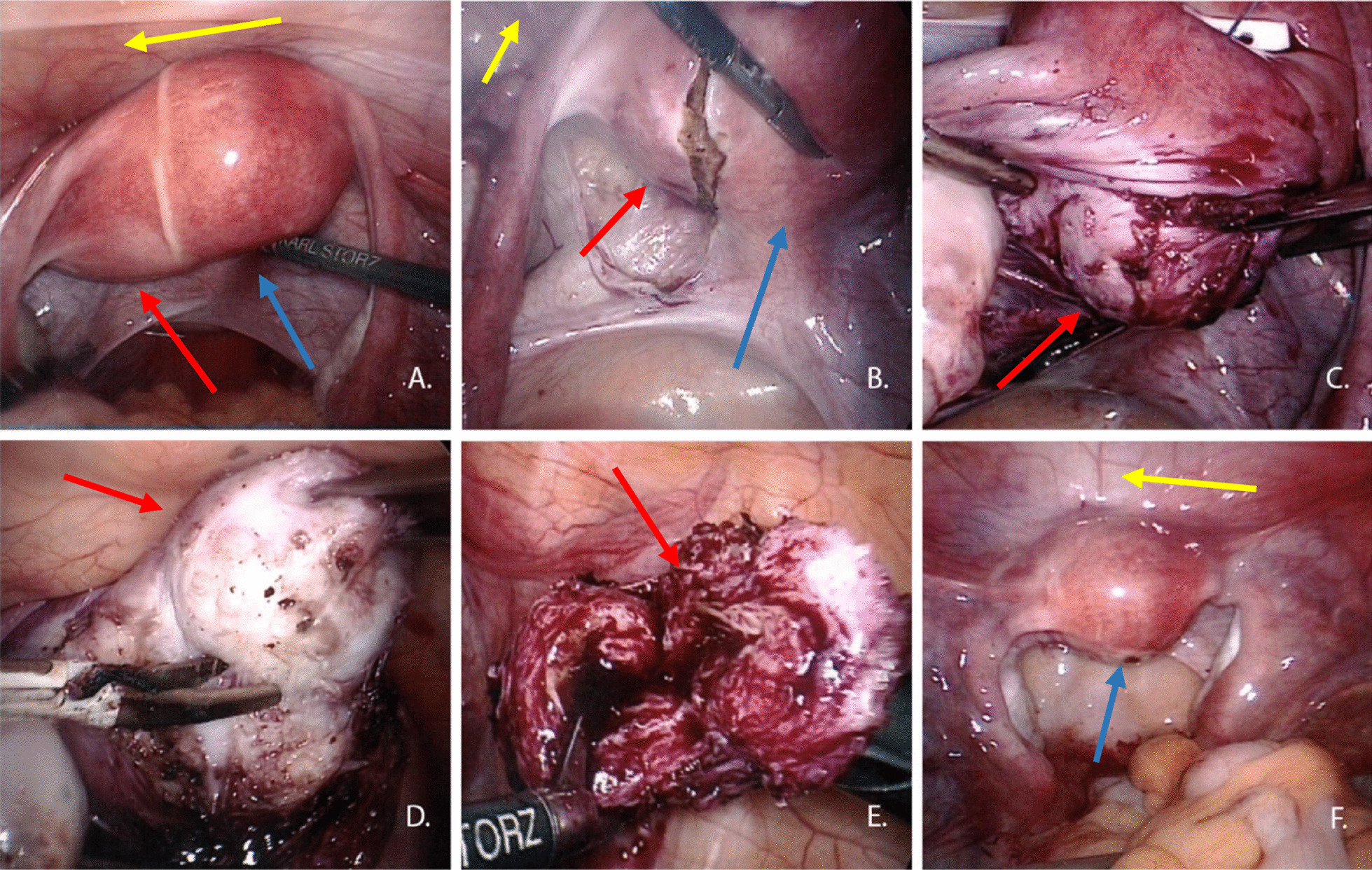
**a** Intraoperative entrance showing the deformation of the uterus because of a rudimentary uterine horn (red arrows mark the rudimentary uterine horn, blue arrow the normal uterus. and yellow arrow the bladder). **b** Intraoperative image. Opening the broad ligament of the uterus and the left parametrium. **c**, **d** Preserving the “parasitic” uterus horn. **e** Opened uterus horn with endometrium. **f** Final intraoperative image after reconstruction of the uterus

Hysteroscopy disclosed a normal cervix with only one uterine cavity, which was symmetrical and presented with two regular tubal ostia. A surgical laparoscopy was subsequently performed. Inspection of the internal genitals revealed normal ovaries and tubes on both sides. There was no evidence of endometriosis. There was a bulge on left side below and lateral to the origin of left fallopian tube from the main uterus causing a slightly asymmetrical shape (Fig. 2a). This spherical protrusion on the left side of the lower corpus area had a diameter of about 3-4 cm. Incision was made over the bulge, just below the insertion of the left tube, as shown in Fig. 2b. The spherical structure could be enucleated without complications from the left parametrium of the uterus while preserving the tubal branch of the uterine artery (Fig. 2c, d). Surprisingly, the left tube arose directly from the uterus, which contrasts with published data about the anatomical position of a rudimentary uterine horn. A chocolate-like secretion appeared after opening the structure. The cavity was found to be lined with endometrium, indicating that the structure was a non-communicating uterine horn (Fig. 2e). After reconstruction of all layers of the uterus, its shape and size were normal, the cavity symmetrical, and the tube outlets on both sides at the regular sites (Fig. 2f).

**Fig. 3:**
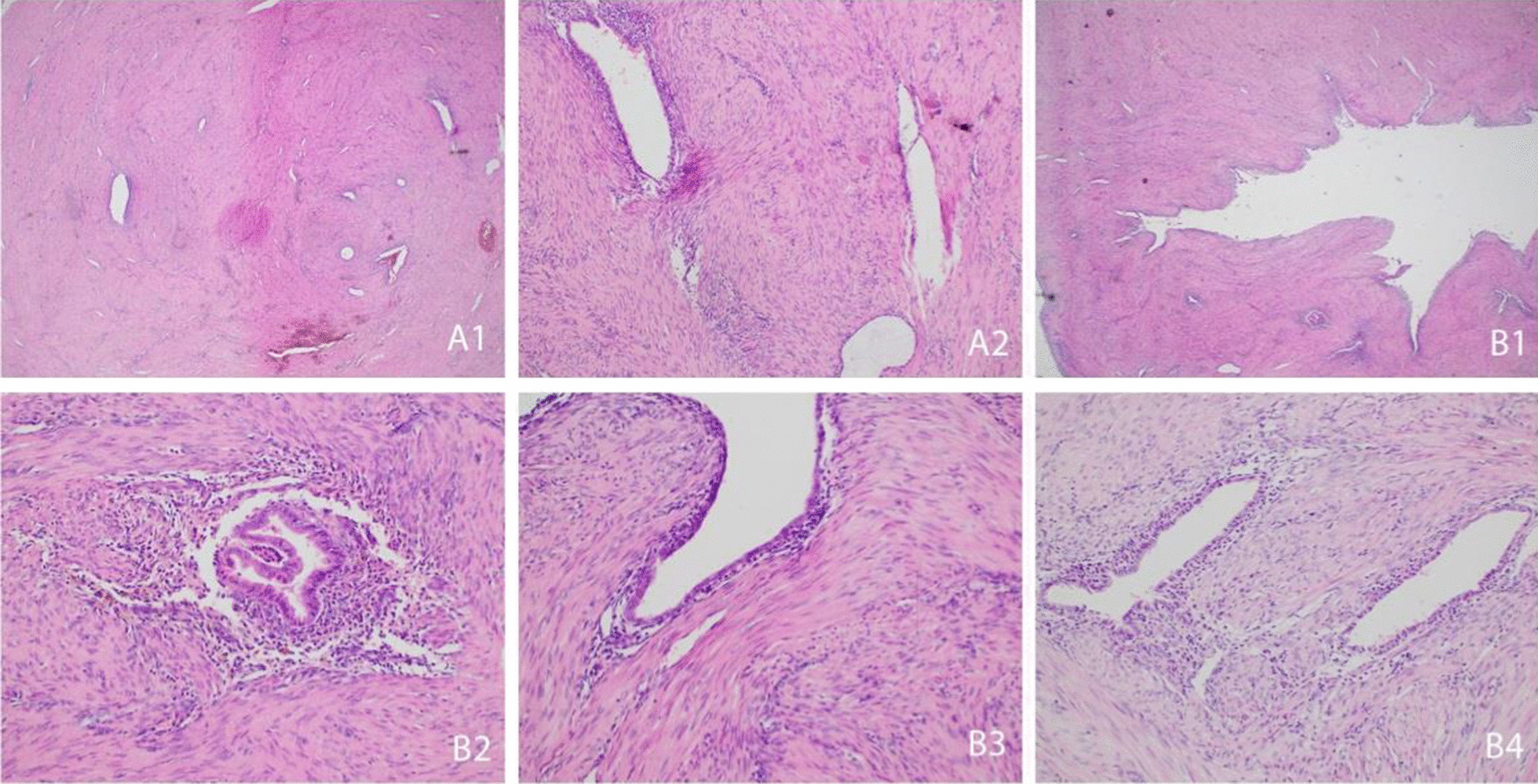
**A1** Overview of the first block. **A2** First block in detail: Irregularly arranged small glands with flat to cuboidal epithelium and uniform nuclei without mitotic activity and sparse compact stromal cells within interlacing bundles of smooth muscle cells. **B1** Overview of the second block: cavity of the uterine horn. **B2**–**B4** Second block in detail: slightly jagged and irregular luminal surface of the endometrial glands with a thin epithelium and cigar-shaped vertically oriented nuclei, sparse intraepithelial neutrophil polymorphs, and mildly pigmented hemosiderin-laden macrophages, corresponding to the macroscopic condition after bleeding

Histopathological investigation revealed an atrophic endometrium and a hyperplastic myometrium, along with a rudimentary uterine horn. It was dumbbell-shaped, measuring 4.0 × 2.6 cm in size and up to 1.2 cm thick, beige-gray-white, partially with small hemorrhagic areas and a partly ambiguous lumen (Fig. 3). Two days after the operation, the patient was discharged in good general health. The follow-up examinations revealed no abnormalities, and the patient had no further symptoms.

## Discussion

We report on a 21-year-old patient suffering from dysmenorrhea, with a non-communicating functioning horn with normal uterine cavity and bilateral fallopian tube. The left fallopian tube arose directly from the cornua of uterus, in orthotopic location, with no connection to the rudimentary uterine horn or structure. This condition is contrary to published data and has not been mentioned in any classification.

The exact rate of Müllerian malformations is unknown because some women with the condition remain asymptomatic and are rarely diagnosed. A meta-analysis revealed a 21-fold higher prevalence of congenital uterine malformations among infertile women than fertile women [11]. Prevalence rates for Müllerian malformations vary greatly from 0.0001% to 10% in patients with symptomatic lower abdominal pain [12, 13].

Müllerian malformations seem to turn symptomatic in adolescence or in the third decade of a woman’s life, but have also been observed at a later age [14]. The most common symptoms of Müllerian anomalies are shown in Table 2. Fedele *et al.* mention that a functional endometrium and hematometra in the non-communicating rudimentary uterine horn is a rare condition [15]. Retrograde menstruation and metaplastic conversion of an omnipotential mesothelium into a functional endometrium is believed to be the cause of symptoms and the development of endometriosis [16]. The anxiety of patients who learn that they suffer from genital abnormalities has been given little attention; psychological factors were shown to affect fertility and are known to be a reason for ambiguous abdominal pain [17].

**Table 2:** Common symptoms of Müllerian anomalies

Clinical condition	Association with Müllerian anomalies
Asymptomatic	1–3.5% [7]
Infertility	6.3% [6]–10% [16]
Ambiguous abdominal pain in adolescence	8.5% [14]
Ambiguous abdominal pain until the age of 50 years	15–20% [38]
Dysmenorrhea	Wide range of reported incidence
Endometriosis	21–31% [15] of patients with a rudimentary uterine horn
Hematomata	6.6 [39]–22.7% [40] of patients with a rudimentary uterine horn

In a study of 266 rudimentary uterine horns, only 26% were identified on transvaginal ultrasound [5]. In another report, the rate of successful diagnosis was higher than 44% [18]. In a further study, the authors reported nearly 100% accuracy when using three-dimensional transvaginal ultrasound. The procedure is promising, but has not yet been established in clinical routine [19, 20]. Three-dimensional computed tomography has also been used for the diagnosis of these malformations [21]. Alborzi *et al.* report that the use of sonohysterography to detect these anomalies eliminates the need for diagnostic laparoscopy [22]. Once a rudimentary uterine horn is suspected, the next step is MRT. The latter investigation remains the gold standard and possesses the greatest sensitivity (as high as 100%) for the detection of these malformations [23]. However, some investigators question the value of magnetic resonance tomography, especially in cases of a septate uterus [24]. The radiologist must be experienced in the diagnosis of gynecological abnormalities.

Patients with a non-communicating rudimentary uterine horn present with cycle-dependent or cycle-independent pelvic pain. Pregnancy in the rudimentary uterine horn occurs in 1 of 76,000 cases [25]. Pregnancy in the cavity of a non-communicating uterine horn is extremely rare, but has been reported in the published literature. It is attributed to transperitoneal migration of spermatozoa [26]. The prevalence of a pregnancy in the cavity of a non-communicating uterine horn is reported to range between 1/140,000 and 1/100,000; in the majority of cases it culminates in a life-threatening rupture [27]. Surprisingly, a few infants were reported to have survived under these circumstances [28].

It is important that appropriate treatment be given not only to symptomatic patients but also to asymptomatic women with a uterine horn containing endometrium, in order to avoid retrograde menses, endometriosis, and adhesions. The gold standard for the treatment of a rudimentary uterine horn is excision by the use of laparoscopy or laparotomy [29]. The rapid advancement of minimally invasive surgery in recent years permits laparoscopic [30] or hysteroscopic treatment in most cases [31]. However, a study comparing reproductive outcomes after abdominal metroplasty for bicornuate uteri reported higher pregnancy rates after 2 years for non-operated patients (95%) than for those who had undergone surgery (84%) [32]. Improved pregnancy rates after metroplasty have been confirmed in other studies as well. Metroplasty also proved more effective in maintaining pregnancy compared to patients who had not undergone surgical treatment [33]. Other methods such as endometrial ablation [34] or surgical connection to the non-communicating uterine horn by the use of hysteroscopy have been reported to be successful [35]. However, in view of the small case numbers treated so far, the procedure should be confined to specialized laparoscopic centers.

Crosby and Hill in 1962 and Musset *et al.* in 1967 proposed their theories about the embryological failure of the duct system leading to uterine malformations [36, 37]. During embryogenesis, a failure of the duct system at various sites may cause a variety of uterine abnormalities. However, the presence of anomalies of the Müllerian ducts in conjunction with a completely normal formation of differentiated end organs of the duct system, as in the present case, is a rare condition and calls for further investigation. The anatomical findings in this case appear to be similar to the second subgroup, namely that of a unicornuate uterus (non-communicating uterine horn). However, there is one difference: in the present case we found a physiological connection between both tubes and the uterine cavity. Our anatomical findings were also not found in the VCUAM (Vagina Cervix Uterus Adnex-associated Malformation) classification [2].

## Conclusion

The present case seems to be a hitherto unprecedented form of an occult non-communicating uterine horn which has neither been categorized in the standard classification [3] system nor reported in the published literature. This was an accidental finding in a case of dysmenorrhea where medical methods failed. However, there are some reports of unusual variants of Müllerian anomalies which resemble this case, but with the important difference that in these cases only a single regular tube was found to be connected to the normal uterine cavity. More cases are needed in order to confirm our hypothesis. Despite the rare incidence of these anomalies, the gynecologist should consider Müllerian anomalies as an important differential diagnosis in women with infertility, abdominal pain, and dysmenorrhea in order to select the appropriate treatment option and help to safeguard and improve the patient’s fertility.

## Data Availability

The datasets used and analyzed during the current study are available from the corresponding author on reasonable request.

## Abbreviations

AMH: Anti-Müllerian hormone
MRT: Magnetic resonance tomography
